# Novel Arsenic Markers for Discriminating Wild and Cultivated *Cordyceps*

**DOI:** 10.3390/molecules23112804

**Published:** 2018-10-29

**Authors:** Lian-Xian Guo, Gui-Wei Zhang, Qing-Qing Li, Xiao-Ming Xu, Jiang-Hai Wang

**Affiliations:** 1 Dongguan Key Laboratory of Environmental Medicine, School of Public Health, Guangdong Medical University, Dongguan 523808, China; glx525@gdmu.edu.cn (L.-X.G.); liqing0752@163.com (Q.-Q.L.); 2 Shenzhen Academy of Metrology and Quality Inspection, Shenzhen 518000, China; zhguiw98@163.com; 3 Guangdong Provincial Key Laboratory of Marine Resources and Coastal Engineering, School of Marine Sciences, Sun Yat-sen University, Zhuhai 519082, China; 4 South China Sea Bioresource Exploitation and Utilization Collaborative Innovation Center, School of Marine Sciences, Sun Yat-sen University, Guangzhou 510006, China

**Keywords:** *Ophiocordyceps sinensis*, *Cordyceps militaris*, arsenic markers, principle component analysis (PCA)

## Abstract

*Ophiocordyceps sinensis* has been utilized in China and adjacent countries for thousands of years as a rare functional food to promote health and treat diverse chronic diseases. In recent years, adulterants are usually identified in the processed products of wild *O. sinensis*. However, the effective adulteration examination has to be additionally performed except their routine test, and accordingly is time- and money-consuming. Recently, arsenic determination has become a necessary test for confirming whether the concentrations of inorganic arsenic are over the *O. sinensis* limit. In this work, the contents of total arsenic and As species in cultivated *O. sinensis, Cordyceps militaris*, and other edible fungi were determined by ICP-MS and HPLC-ICP-MS. The results suggest that the As speciation exhibits a species-specific behavior, and accompanies the effect of the As background. The proportions of unknown organic As and contents of total As may be considered as sensitive markers for discriminating wild *O. sinensis*. This result provides a novel clue for discriminating wild and artificially cultivated mushrooms/their products, with emphasis on arsenic markers for authenticating wild *O. sinensis*.

## 1. Introduction

**Figure 1:**
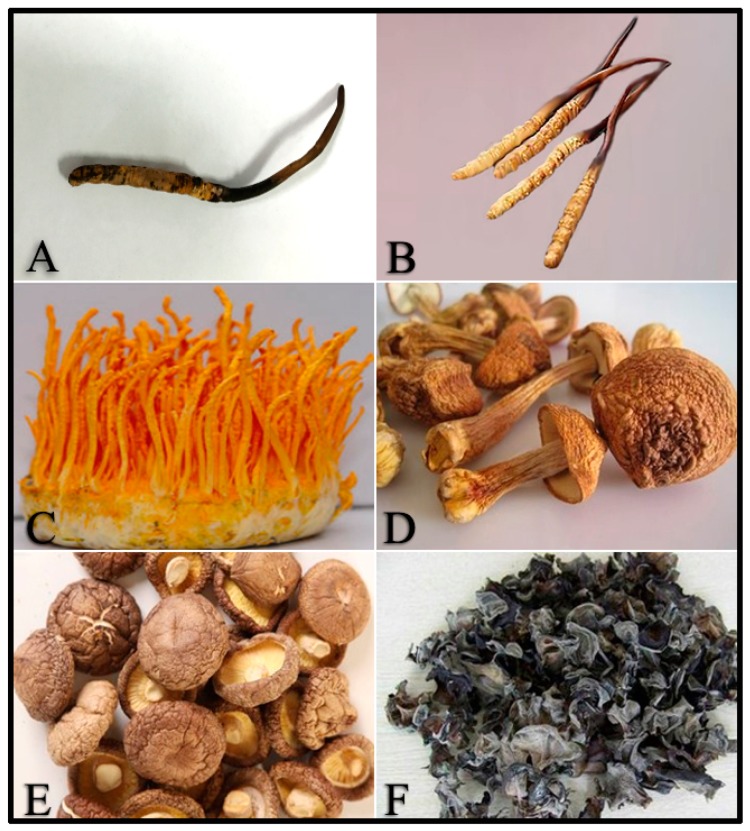
Morphological characters of the samples for this study. (**A**) wild *Ophiocordyceps sinensis*, (**B**) cultivated *Ophiocordyceps sinensis*, (**C**) *Cordyceps militaris*, (**D**) *Agaricus blazei*, (**E**) *Lentinus edodes*, and (**F**) *Auricularia auricula*.

*Ophiocordyceps sinensis* [1] (Figure 1A, syn. *Cordyceps sinensis*), a mysterious entomogenous fungus distributed on the Qinghai-Tibetan Plateau, is popularly referred as winter-worm-summer-grass (Dong Chong Xia Cao in Chinese) [2,3,4,5]. *O. sinensis* has been utilized in China and adjacent countries for thousands of years as a rare functional food to promote health and treat diverse chronic diseases [2]. In 1993, *O. sinensis* attracted worldwide attention because Chinese women athletes broke several world records in running events at the National Games, and the meritorious performances were later attributed (at least in part) to the consumption of this fungus [6]. Subsequently, modern pharmaceutical studies have increasingly shown that it has multiple remarkable functions such as anti-tumor, anti-inflammatory, nephroprotective, antioxidant, antihyperglycemic, anti-apoptosis, immunoregulatory, and hepatoprotective [7,8].

Correspondingly, the significant medicinal functions have resulted in a large demand for wild *O. sinensis* and sharp increase of the retail price (15,000 USD/kg for medium quality) in recent decades. To satisfy its market need, cultivated *O. sinensis* (Figure 1B) [9,10] (and multifarious processed products of wild *O. sinensis* have rapidly emerged and widely merchandised [11], with the presence of adulterated products made from its cheap substitutes. To standardize the *Cordyceps* market, the most urgent problem is to establish the effective indicators for authenticating *O. sinensis*-related products. Traditionally, the morphology, color and odor have been applied to effectively authenticate wild *O. sinensis* [12], due to the direct consumption of the whole fungus-larva complex (Figure 1A) such as preparing medicinal soups or liquors. However, these routine methods are insufficient to discriminate wild and cultivated *O. sinensis* (Figure 1B) [9,10] because they are exactly identical in these aspects. Furthermore, the health foods related to wild *O. sinensis* have become increasingly popular in the convenient forms such as tablet, capsule and oral liquid [13], and the above-mentioned empirical methods have been unfeasible for adulteration test. Recently, HPLC, bio-molecular fingerprint and isotopic tracing seem to be appropriate methods for this test [14,15,16,17]. As a case study, our group has proved that the stable carbon isotope ratio may be an effective indicator for authenticating the products of wild *O. sinensis* [17].

In 2016, the China Food and Drug Administration (CFDA) revealed that the high contents of total As (4.4–9.0 mg/kg) [18], nearly five times the limit value of 1 mg/kg for functional foods (GB16740-2014) [19], were detected in *O. sinensis*. Soon afterwards, CFDA ordered that all of the pilot works of *O. sinensis* as a functional food be discontinued [20]. This event caused anxiety in the functional food market, and prompted to consider the concentrations of total As and iAs as two obligatory test items for wild *O. sinensis* and its processed products. In our latest study [21], we found that the contents of total As were massive (4.00–5.25 mg/kg), but iAs was minor (As^III^, 4.1–6.0% and As^V^, 1.3–3.2%) and oAs (MMA, DMA, and AsB) was absent or trace in *O. sinensis*. Further, we surprisingly found that As species were consistent with each other among different bulk samples even if the samples came from different producing areas [21]. Thus, As species may be used as indicators to discriminate both wild *O. sinensis* and its substitutes or adulterants.

To establish novel arsenic markers for discriminating wild *O. sinensis*, we comparatively studied the total As and As species in wild *O. sinensis* (WOS, Figure 1A) and its common substitutes, including cultivated *O. sinensis* (COS, Figure 1B), cultivated *Cordyceps militaris* (CM, Figure 1C) [22], and other mushrooms [*Agaricus blazei* (AB, Figure 1D), *Lentinus edodes* (LE, Figure 1E), and *Auricularia auricula* (AA, Figure 1F)]. These data were scrutinized by principal component analysis, and several sensitive arsenic markers were developed for discriminating wild *O. sinensis*. Thus, this study provides a novel clue for distinguishing wild and artificially cultivated mushrooms/their processed products in the routine inspection of hazardous materials.

## 2. Results

### 2.1. Total Arsenic

**Figure 2:**
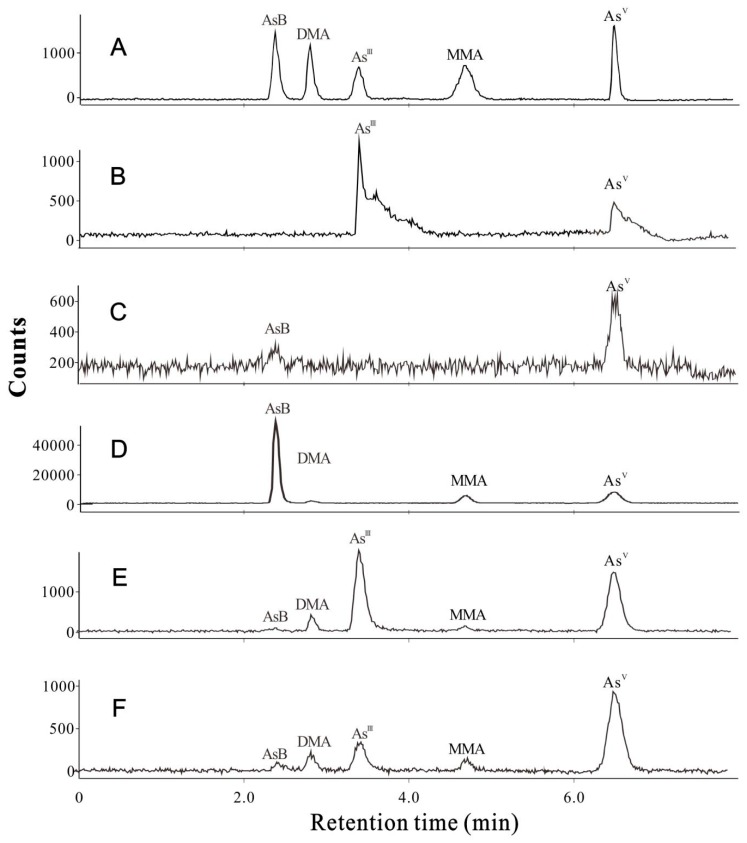
Total ion currents of As species in the standards (**A**), COS (**B**), CM (**C**), AB (**D**), LE (**E**), and AA (**F**).

The quality control for both total As and As species analyses in this study was validated by determining the linearity (*R* = 0.9997–1.000) (Table S1), limits of detection (LODs < 2 μg/kg) (Table S1), limits of quantification (LOQs < 6 μg/kg) (Table S1), relative standard deviations (RSDs < 7%) (Table S2), the recoveries (92.0–106.0%) (Table S3), CRM materials (Table S4), and extract efficiency (87.0–104.5%) (Table S5). The total ion currents of As species in standards (A), COS (B), CM (C), AB (D), LE (E), and AA (F) are presented in Figure 2, and the standard adding tests are illustrated in Figures S1–S5.

**Figure 3:**
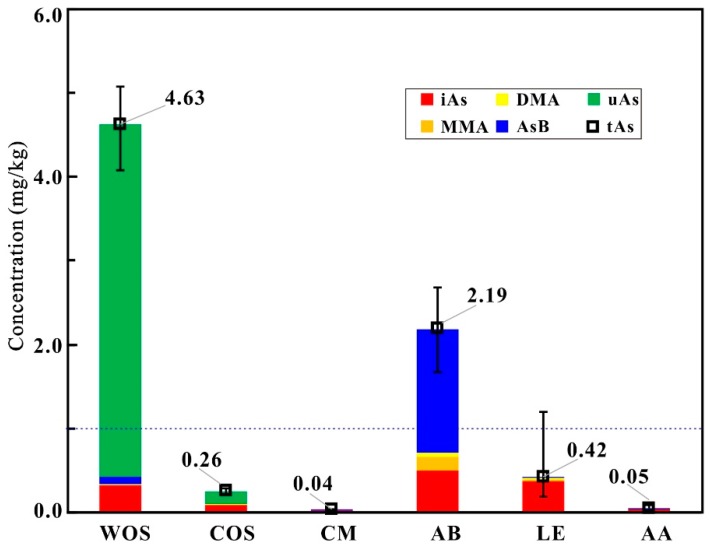
Average concentrations (mg/kg dry weight) of total As and As species in the samples. The columns in red, orange, yellow, blue, and green denote iAs, MMA, DMA, AsB, and uAs, respectively. The blue dashed line denotes the arsenic limit for functional foods.

The contents of total As in samples are presented in Table 1 and Figure 3. It can be seen from Table 1 that the levels of total As in COS, CM, AB, LE, and AA samples are at the intervals of 0.223–0.289 mg/kg, 0.036–0.047 mg/kg, 1.680–2.677 mg/kg, 0.188–1.203 mg/kg, and 0.028–0.087 mg/kg, respectively. The concentrations of total As exhibit significant differences (*p* < 0.05) among different sample groups. In terms of the means of total As (Figure 3), WOS [21] > AB > 1 mg/kg (the limit for functional foods highlighted in the blue dashed line in Figure 3, GB16740-2014 [19]) > LE (except two LE samples slightly beyond the limit) > COS > AA > CM.

### 2.2. Arsenic Species

**Figure 4:**
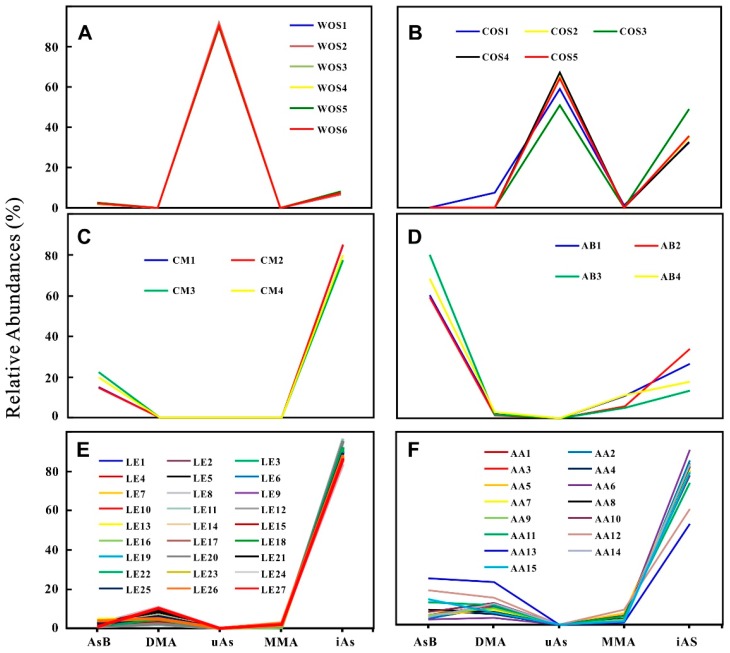
Variation of the relative abundances (%) of As species in wild Ophiocordyceps sinensis (**A**), cultivated Ophiocordyceps sinensis (**B**), Cordyceps militaris (**C**), Agaricus blazei (**D**), Lentinus edodes (**E**), and Auricularia auricula (**F**).

The abundances of As species in samples are presented in Table 1 and Figure 3. It can be seen that the contents of iAs in the COS samples are at the interval of 0.078–0.140 mg/kg (32–49%); while AsB, DMA and MMA are trace in most of the samples. Similar with WOS [21], the COS samples additionally have a portion of uAs with the range of 0.145–0.180 mg/kg (51–67%). The As species in CM, LE, AA, and AB are mostly confined to five As species for the routine detection; and the average relative abundances of AsB, DMA, MMA, and iAs change at the intervals of 2–67%, 0–9%, 0–8%, and 23–90%, respectively. The abundances of As species evidently vary in different sample groups (Figure 3), i.e., oAs predominant in WOS, COS, and AB; uAs rich in WOS and COS; the contents of AsB high in AB; iAs dominant in CM, LE, and AA. Figure 4 intuitively exhibits the similar profiles of As species variations in the same group but the different ones in different groups.

According to their profiles of As species, all of the studied samples may be divided into three types. Type 1 (Figure 4A,B): WOS and COS have the highest peak at uAs (Based on the chromatography behaviors under anion exchange HPLC-ICP-MS and the stability under the H_2_O_2_ treatment, it may be an arsenosugar, which is frequently reported to be co-eluted with DMA, AsB, MMA, and As^III^ under anion exchange HPLC-ICP-MS [23]. More detailed analyses were reported in our previous work) [21] and a lower peak at iAs. Unlike common mushrooms cultivated on plant-based substrates [24], WOS and COS belong to a kind of the fungus-larva complex, which may contain a large part of oAs in the animal-based sclerotium, and accordingly possess the completely different profiles. Type 2 (Figure 4C,D): CM and AB have two peaks at AsB and iAs. AsB is the major As species in AB, which is consistent with the previous report for the store bought *A. bisporus* [24]. This result indicates that AB in the food market may be safely consumed although its total As is significantly higher than the permitted limit. AsB, an osmolyte commonly distributed in marine organisms in response to salinity changes, is also considered as an osmolyte in certain mushrooms to maintain the fruiting body structures and specific morphologies [24]. Although there is no report on AsB in mushrooms with rod-like stromata, we still infer that AsB in CM may be related with its rod-like stroma on the basis of the above theory. Type 3 (Figure 4E,F): LE and AA possess a slightly peak at DMA and a sharp peak at iAs. A previous study [24] suggested that the relative abundance of iAs in LE was approximately 40% of the total As, and the remaining over 30% As was not extracted for As species analysis. In this study, the extraction efficiencies were very high (87.0–104.2%), and the proportions of iAs reached 90% on average. The comparative analysis indicates that the As for species analysis was extracted by 2% HNO_3_ at 70 °C for 2 h in reference 24; while the As was extracted at 90 °C for 12 h and shaken frequently using the same solvents in this study. Thus, we consider that our procedures for extracting As species are more proper, and the unextracted As shown in reference 24 may be iAs in comparison with the iAs proportions.

## 3. Discussion

### 3.1. Arsenic Fingerprint for Adulteration Test

The species and abundances of metabolic products of hazardous materials in organisms might be species-specific. To ensure the survival and reproduction of organisms, each of them can develop a detoxifying strategy to withstand hazardous materials, which sneak into their body from environments [25,26]. Arsenic as a common element in the ecosphere can be transferred in organisms via their trophic chain. Accordingly, As is naturally accumulated in sea foods [27,28], land animals [29], terrestrial crops [30], algae [31], and mushrooms [32]. Therefore, As is one of the most concerned hazardous materials over the world due to its toxicity. The toxicity of As depends on its chemical forms. The iAs species, such as arsenite (As^III^) and arsenate (As^V^) are the most toxic, and most of them exist in soils and water. When entering organisms, iAs begin to be transformed to low toxic oAs species in vivo. The initial metabolic products are MMA and DMA, which are much less toxic than iAs to human beings; and their subsequent metabolic products are other non-toxic oAs complexes such as AsC, AsB, arsenosugars, and arsenolipids [33]. Thus, the As conversion in organisms is generally considered as the detoxification of iAs. Both the species and abundances of As metabolic enzymes vary among different organisms [33], and subsequently result in the variability of oAs products among them. Correspondingly, the species and abundances of oAs exhibit a species-specific trend.

Previous studies revealed that the contents of As species varied significantly in different mushrooms [34]. The studies of As species for store-bought mushrooms (usually grown in the low As background) suggested that several mushrooms contained high oAs, e.g., *Leccinum scabrum*, *Paxillus involutus*, and *Agaricus bisporus*; and some mushrooms, e.g., *Agaricus balieimurrill*, *Leucopaxillus giganteus*, *Pleurotas eryngii*, and *Lepista nuda* evidently enriched toxic iAs, such as As^III^ and As^V^ [35]. The As species in mushrooms from the high As background indicated that some of them mainly contained oAs, e.g., DMA (approximately 70% of total 1420 mg/kg As) in *Laccaria amethystina* from a heavily polluted site was the highest proportion [36]; the As in *Collybia butyracea* collected near an As smelter consisted of 8.8 mg/kg AsB and 1.9 mg/kg DMA, with the total As content of 10.9 mg/kg in the fruiting body [37]; and some mushrooms contained toxic iAs, such as As^III^ and As^V^ [38]. Although the mechanisms of uptake, retention and biotransformation of arsenic by mushrooms are still not clear, every mushroom often contains similar arsenicals regardless of the harvest location, suggesting that the mushrooms have an ability to metabolize arsenic compounds [24,39,40]. In addition, it has been theorized that only the more highly evolved fungi (e.g., *Geastrumand agaricus*) can produce more complex arsenic compounds [40]. In this study, Figure 4 presents the variation in the relative abundances of MMA, DMA, AsB, uAs, and iAs among different groups; and all of the curves coincide within the same group. This coincidence may be caused by the same As metabolic ability within the same biological variety.

However, there is the evident difference in the relative abundances of As species between WOS (Figure 4A) and COS (Figure 4B). Clearly, the profiles of As species are not coincident with each other even if they belong to the same species. Furthermore, the total As in WOS is tenfold than that in COS (Figure 3). This discrepancy implies that As species are also influenced by other factors. For wild *O. sinensis*, as a special organism occurred in the Qinghai-Tibetan soil ecosystem, the As species and their concentrations were derived from the complicated synergy among soils (living environment and alternative foods of the host larvae [3]), plants (favorite foods of the host larvae), host larvae and *O. sinensis* fungus [4,5]. The average arsenic concentration in the Tibetan soils was 18.7 mg/kg (*n* = 205) [41], and evidently higher than the average value of the upper continental crust, 1.5 mg/kg [42]. The As in soils could be firstly accumulated into plants via their roots, and then was gradually amplified in the host *Thitarodes* larvae, which utilized the tender plant roots as their favorite foods for approximately 3 years [3,4], and accumulated a tremendous amount of As from the high As background in the duration of their life cycle. Thus, the total As in wild *O. sinensis* were plentiful, not only from the high-As host larvae as its substrate but also from the residual As in its larva-derived sclerotium. As for COS, it was cultivated in the artificially-controlled circumstance instead of the high As background in the Qinghai-Tibetan Plateau. The host larvae were fed with the artificial diet on the optimal temperature and soil conditions for promoting their growth [43] and lowering the cost of breeding time (less than a year to develop to the susceptible instars, which is greatly shorter than that in the wild [9]). Therefore, the low As background and shorter duration of the life cycle result in low As accumulation in COS. In addition, the exposure time of arsenic in the environment may also play a role in arsenic transformation [44]. Different duration of the life cycle may also induce different As metabolic processes and efficiencies in the host larvae, and ultimately result in the differentiation of As composition in COS.

*Cordyceps militaris* as a fungus-pupa complex is evidently different from WOS and COS occurred as a fungus-larva complex; and its stromata are usually consumed as edible parts [22]. The stromata of *C. militaris* were massively cultivated on the artificial substrates in food industries, and also brisked up in food markets [45], just like *C. militaris* (CM) in this study. The analysis of total As in CM is usually neglected owing to the low As abundances in substrates. Similarly, the contents of total As in the two cultivated edible mushrooms, LE and AA are also low due to the low As abundances in their artificial substrates.

When *A. bisporus* was cultivated in the control substrate (low As background, 3.8 ± 0.2 mg As/kg), the concentrations of total As were 0.50 0.03 mg/kg, and the most abundant speciation was AsB (0.31 ± 0.08 mg/kg, about 68.4%) [38]. In this study, *A. bisporus* was wild and grown on the natural substrate. Although the contents of total As (1.680–2.677 mg/kg) are evidently higher than those in the controlled *A. bisporus*, the most abundant speciation was identical (AsB, 59 80%). The above consistency can be explained by the species-specific speculation [24]. However, when cultivated in an iAs seriously contaminated substrate (1000 mg As^V^/kg), *A. bisporus* enriched iAs (22.8 ± 1 mg/kg; 98% of the total As) [38]. In this situation, most of the As in *A. bisporus* occurred in the highly toxic iAs form, and was not transformed into AsB. The same phenomenon was discovered in some marine organisms, and considered to be attributed to that the biological pathways became saturated at the higher As concentrations [46]. Therefore, higher concentrations may have inhibited the microbial transformation of As [44]. This is to say, if the background is high in iAs, the excessive iAs will be passively transferred into mushrooms from the substrate, but cannot be converted into oAs via the fungal transformation of As [38].

### 3.2. Novel Arsenic Markers for Discriminating Wild and Cultivated Cordyceps

**Figure 5:**
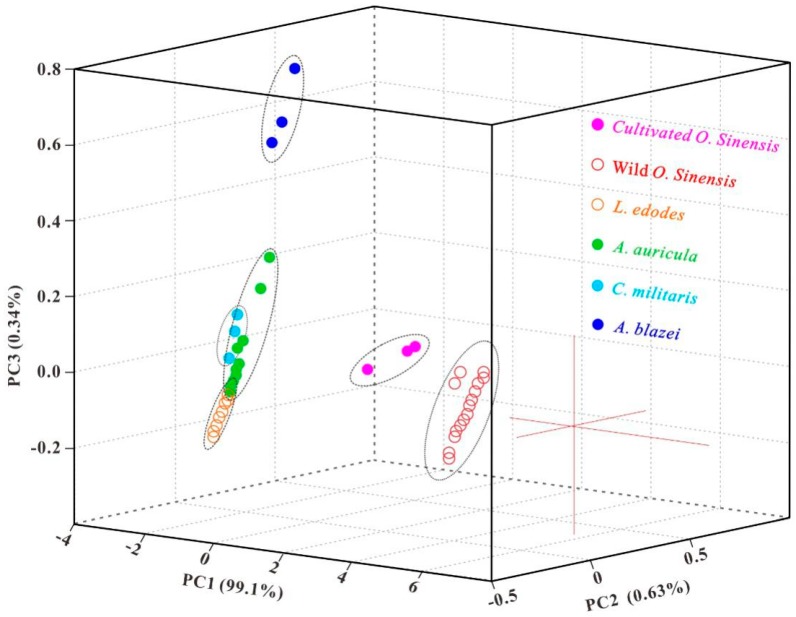
Three-dimensional PCA score plot for discriminating six sample groups. The first three principal components are displayed, accounting for 99.01%, 0.63%, and 0.34% (99.98% in total) of the total variation, respectively.

Since the As-accumulated mushrooms and *O. sinensis* are frequently reported by CFDA, the detection of total As and five As species has become the routine test items for edible mushrooms and *O. sinensis*. In view of As composition dependent on biological species and As backgrounds, we may adopt As species and their abundances as markers for authenticating wild *O. sinensis*. In other words, the routine food safety inspection can be further utilized as an adulteration test. The discrepancy of total As (Figure 3) and As species in abundance (Figure 4) between wild *O. sinensis* and the other groups confirms the above speculation. To validate the possibility, the PCA scores from the data (Table 1 and Table S6) of total As and As species were examined, and the first three principal components displayed in a three-dimensional plot (Figure 5), accounting for 99.01%, 0.63%, and 0.34% (99.98% in total) of the total variation, respectively. The detailed ranges for every group were presented in Table 2. Samples in each group were classified into discrete clusters, indicating that PCA could be used to distinguish one group from another. From the PC1 axis (−2.80 to 6.55), WOS in the range of 1.40–6.55 was well separated from the other groups with the positive scores. From the PC2 axis (−0.60 to 0.80), COS at the interval of 0.45–0.80 was well discriminated from the remaining five groups with the gap of −0.40 to 0.15 on this axis. Thus, the As composition can be applied to discriminate between wild/cultivated *O. sinensis* and its substitutes. In addition, from the PC3 axis (−0.20 to 0.95), AB samples (0.55 to 0.95) can also be distinguished from the other samples (−0.20 to 0.35) closely distributed on the PC3 axis.

**Figure 6:**
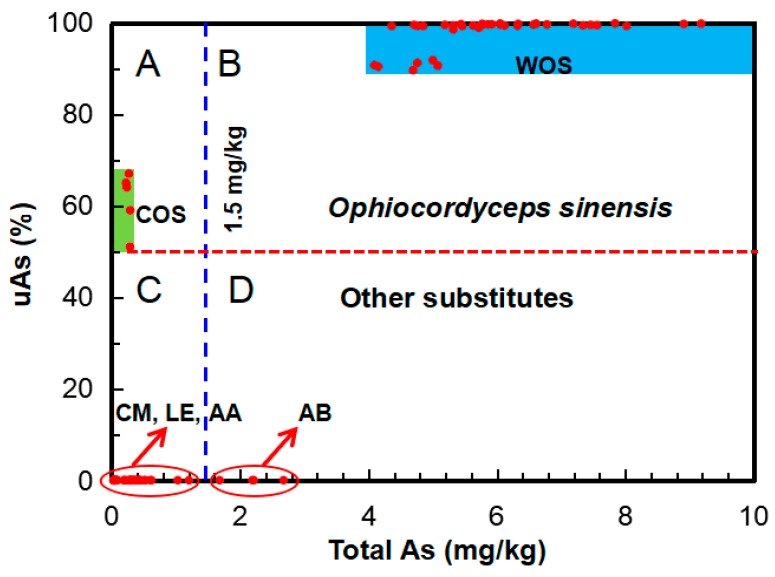
uAs (%) vs. tAs (mg/kg) plot illustrating the fields of wild *O. sinensis* [blue in field (**B**)], cultivated *O. sinensis* [green in field (**A**)], and the other mushrooms. Red dashed line divides the samples into the groups of *O. sinensis* (WOS and COS) and the groups of the other mushrooms (CM, AB, LE, and AA). Blue dashed line divides the samples into the groups of wild (WOS and AB) and cultivated (COS, CM, LE, and AA) mushrooms.

In this study, to discriminate wild *O. sinensis*, the abundances of total As and uAs can be applied as sensitive markers. Although the uAs species are still not clear, we consider that they may belong to asenosugars on the basis of the H_2_O_2_ test and their chromatographic behaviors in our previous study [21]. They were mostly formed by long-term metabolic processes in the host larva. Thus, the larva-originated sclerotium in WOS and COS may contribute to most of the uAs, which can be subsequently used to distinguishing WOS and its substitutes. In this study, we may construct the uAs (%) vs. total As (mg/kg) plot (Figure 6) from the data (Table 1 and Table S6) for discriminating adulteration. It can be seen from Figure 6 that the plot clearly exhibits different fields (A, B, C, and D) for different sample groups, i.e., *O. sinensis*, whatever WOS (total As, 4.1–9.2 mg/kg; uAs, 89.6–100%) or COS (total As, 0–0.29 mg/kg; uAs, 59 67%), dropped in field A or B (above the red dashed line, uAs > 59%); the other mushrooms (CM, AB, LE, and AA) except *O sinensis* in the field below the red dashed line; wild mushrooms (including WOS and AB) in fields B and D in the right side of the blue dashed line (1.5 mg/kg [47]); the other cultivated mushrooms (CM, LE, and AA) in the left side of the blue dashed line. Thus, Figure 6 can be evidently used to discriminate wild/cultivated *O. sinensis* from its substitutes. If the As composition of one *Cordyceps* product drops in the blue field, it may be derived from wild *O. sinensis*.

It should be pointed out that the above-mentioned As markers can be used to authenticate not only wild *O. sinensis* from its substitutes/processed products, but also wild edible fungi from their cultivated ones (Figure 6), because the As contents in cultivated fungi are generally low (*A. bisporus*, 0.19–1.50 mg/kg), especially wood-cultivated fungi (*Pleurotus ostreatus*, 0.09–0.50 mg/kg; *L. edodes*, 0.04–0.07 mg/kg) [47]. To reduce the chances of false positives or false negatives, more accurate As indicators and ranges need to be explored and ascertained on the basis of the more data in future.

## 4. Materials and Methods

### 4.1. Reagents and Standards

All the solutions were prepared by deionized water (Millipore, Bedford, MA, USA). Anhydrous sodium acetate, potassium nitrate, sodium dihydrogen phosphate, and disodium ethylenediaminetetraacetate (HPLC grade, J & K Chemical, Beijing, China) were used for chromatographic mobile phases. Concentrated HNO_3_ (69%) was used to digest samples for determining the contents of total As; and dilute HNO_3_ (2%) was adopted to extract As species from samples. Standards for measuring both total As (10 mg As/L) and As species (As^III^, 0.233 μmol/g; As^V^, 1.011 μmol/g; MMA^V^, 0.355 μmol/g; DMA^V^, 0.706 μmol/g; and AsB, 0.518 μmol/g) were obtained from diluting and compounding the standard stock solutions (The National Institute of Metrology, Beijing, China). All stock solutions were stored at 4 °C; while dilutions used for analysis were prepared daily. The following National Standard Reference Materials were used in this study: GBW10049 (Green Chinese Onion, Institute of Geophysical and Geochemical Exploration, Langfang, China), GBW10051 (Pork liver, Institute of Geophysical and Geochemical Exploration, Langfang, China), GBW08573 (Yellow fin tuna, the Second Institute of Oceanography, Hangzhou, China), and GBW(E)100358 (Rice, the National Analysis Center for Ion and Steel, Beijing, China).

### 4.2. Samples

Twenty-five samples of cultivated *O. sinensis* (COS, Figure 1B) were freshly purchased from the Horizon East Company (Shenzhen, China), and then freeze-dried in laboratory. To ensure the enough quantity for subsequent As analysis, five pieces of COS were combined into each sample, which was named as COS 1, 2, 3, 4 or 5, respectively. These samples were individually pulverized with the size of less than 40 meshes, sealed in the plastic bags, and stored at 4 °C. Cultivated *Cordyceps militaris* (CM, Figure 1C), wild *Agaricus blazei* (AB, Figure 1D), cultivated *Lentinus edodes* (LE, Figure 1E), and cultivated *Auricularia auricula* (AA, Figure 1F) were obtained from a food market (Shenzhen, China). Referring to the procedures of treating *O. sinensis*, we prepared these samples for As analysis.

The methods for sample digestion and As determination followed the previous reference [21]. Briefly, for total As analysis, approximately 500 mg of each powdered sample was mixed with 20 mL concentrated HNO_3_; and digested in a microwave-assisted system with the following procedures: (1) heated to 120 °C in 5 min and held at 120 °C for 5 min; (2) 150 °C in 5 min and held at 150 °C for 5 min; (3) 170 °C in 5 min and held at 170 °C for 5 min; (4) 190 °C in 5 min and held at 190 °C for 20 min; and (5) cooled to room temperature. For As species analysis, 1 g of each powdered sample was used to extract As species with 20 mL dilute HNO_3_ in a 90 °C water bath for 12 h, and shaken on a vortex for 1 min every 2 h. All of the digested products were centrifuged and filtered; and the final extracts were kept at 4 °C before analysis.

### 4.3. Total Arsenic Analysis

Total As analysis was conducted according to our previously-reported method [21]. The filtered supernatant was replenished with water up to 25 mL, and subjected to an Agilent 7800 ICP-MS (Agilent Technologies, Palo Alto, CA, USA). The analytical conditions were described as below: RF power, 1550 W; carrier gas, 1.03 L/min; dilution gas, 0.10 L/min; collision mode; He flow rate, 4.3 mL/min; plasma gas flow rate, 15 L/min; auxiliary gas flow rate, 0.9 L/min; and selected isotope, *m*/*z* 75. The concentrations of total As in samples were quantified through a As^V^ standards calibration curve (Calibration points: 5, 10, 50, 100, and 200 ppm). The corresponding digestion blanks and total As in CRMs were measured on the basis of the current method.

### 4.4. Arsenic Speciation Analysis

Analyses of As species (As^III^, As^V^, MMA^V^, DMA^V^, and AsB) were conducted according to our previously-established method [21]. Briefly, As species were separated by an Agilent 1260 HPLC (Agilent Technologies, Palo Alto, CA, USA), which was equipped with an autosampler, a guard column (IonPac AG19, 4 mm × 50 mm), and a separation column (IonPacAS19, 4 mm × 250 mm). The chromatographic conditions were described as below: mobile phase, a mixed solution of 10 mmol/L anhydrous sodium acetate, 3 mmol/L potassium nitrate, 10 mmol/L sodium dihydrogen phosphate, and 0.2 mmol/L disodium ethylenediaminetetraacetate buffer; flow rate, 1.0 mL/min; column temperature, 25 °C; injection volume, 50 μL. The separated As species were determined by ICP-MS, and identified and quantified by a calibration curve of the standards (Calibration points: 0, 2.5, 5, 10, 50, 100, and 200 ppb). To correct the matrix effects which might result in the shift of retention time, considerable amounts of internal standards (2.5 ppb for COS, 5 ppb for CM, and 5 ppb for AA; while for AB, which contains a large amount of AsB, 100 ppb of the standards were firstly added in AB, and then diluted with 2% HNO_3_ to the ratio of 1:10) were added in each sample. Due to the complexity of the sample matrix, some of the oAs species, other than the above three oAs species (MMA^V^, DMA^V^, and AsB) might exist in *O. sinensis*, and they were measured using the method reported in our previous study [21]. To check the extraction efficiency for As species analysis, the concentrations of total As in the extracts of As species digested by dilute HNO_3_ were directly measured by ICP-MS; and then compared with the extracts of total As treated by concentrated HNO_3_. Extraction blanks were also determined in each work session, and the iAs species in CRMs were determined using this method.

### 4.5. Statistical Analysis and Data Processing

Statistical analysis was conducted using the IBM SPSS Statistics ver. 20 (Microsoft Corp., Redmond, WA, USA). The total As contents and As species were expressed as their average values of three determinations with the relative standard deviation (RSD) of less than 7%. Principle component analysis (PCA) was used to discriminate different sample groups. Based on the Fuzzy C-means (FCM) method, the three-dimensional PCA score plot was drawn by MATLAB ver. 7.0.4 (MathWorks Inc., South Natick, MA, USA).

## 5. Conclusions

ICP-MS and HPLC-ICP-MS have been employed to measure both total As and As species in wild and cultivated *O. sinensis* as well as multiple edible fungi. The results indicate that both total As and As species exhibit the species-specific behavior and the effect of the As background. The proportions of uAs and the contents of total As are considered as effective markers for discriminating wild *O. sinensis* from its substitutes/processed products.

## 6. Patents

A new method for discrimination of wild and cultivated *Ophiocordyceps sinensis*. Patent No. 201810564330.1

## Figures and Tables

**Table 1:** Concentrations and percentages of arsenic species in all the detected samples.

Samples	AsB mg/kg (%)	DMA^V^ mg/kg (%)	MMA^V^ mg/kg (%)	iAs mg/kg (%)	uAs mg/kg (%)	Total As mg/kg
COS1	BDL	0.022 (7)	0.003 (1)	0.093 (32)	0.171 (59)	0.289
COS2	BDL	BDL	BDL	0.078 (35)	0.145 (65)	0.223
COS3	BDL	BDL	BDL	0.140 (49)	0.145 (51)	0.285
COS4	BDL	BDL	BDL	0.087 (33)	0.180 (67)	0.267
COS5	BDL	BDL	BDL	0.084 (36)	0.152 (64)	0.236
Mean_COS_	BDL	0.004 (1)	BDL	0.096 (37)	0.158 (51)	0.260
LE1	0.006 (2)	0.017 (5)	0.008 (2)	0.343 (92)	BDL	0.375
LE2	0.004 (1)	0.018 (7)	0.006 (2)	0.255 (90)	BDL	0.284
LE3	0.007 (2)	0.019 (6)	0.005 (2)	0.262 (90)	BDL	0.292
LE4	0.007 (2)	0.032 (8)	0.010 (2)	0.339 (88)	BDL	0.387
LE5	0.004 (1)	0.031 (8)	0.006 (1)	0.377 (90)	BDL	0.419
LE6	0.004 (1)	0.009 (3)	0.005 (1)	0.327 (95)	BDL	0.344
LE7	0.018 (1)	0.063 (5)	0.015 (1)	1.107 (92)	BDL	1.203
LE8	0.008 (3)	0.032 (11)	0.009 (3)	0.248 (84)	BDL	0.296
LE9	0.005 (2)	0.018 (6)	0.006 (2)	0.251 (90)	BDL	0.280
LE10	0.007 (2)	0.035 (10)	0.009 (2)	0.304 (86)	BDL	0.354
LE12	0.005 (1)	0.012 (2)	0.004 (1)	0.597 (97)	BDL	0.618
LE13	0.006 (1)	0.019 (4)	0.009 (2)	0.406 (92)	BDL	0.440
LE14	0.028 (5)	0.031 (5)	0.012 (2)	0.529 (88)	BDL	0.600
LE15	0.007 (2)	0.026 (9)	0.006 (2)	0.262 (87)	BDL	0.301
LE16	0.007 (2)	0.018 (4)	0.008 (2)	0.413 (92)	BDL	0.447
LE17	0.021 (2)	0.050 (5)	0.007 (1)	0.952 (92)	BDL	1.029
LE18	0.006 (2)	0.016 (5)	0.007 (2)	0.285 (91)	BDL	0.315
LE19	0.009 (2)	0.027 (6)	0.010 (2)	0.387 (90)	BDL	0.432
LE20	0.004 (1)	0.013 (3)	0.006 (1)	0.487 (96)	BDL	0.510
LE21	0.007 (2)	0.027 (9)	0.007 (2)	0.273 (87)	BDL	0.314
LE22	0.009 (2)	0.025 (5)	0.008 (2)	0.483 (92)	BDL	0.525
LE23	0.004 (2)	0.014 (7)	0.003 (2)	0.177 (89)	BDL	0.198
LE24	0.007 (2)	0.024 (7)	0.007 (2)	0.317 (89)	BDL	0.355
LE25	0.003 (2)	0.012 (6)	0.004 (2)	0.168 (89)	BDL	0.188
LE26	0.008 (4)	0.011 (5)	0.006 (3)	0.190 (88)	BDL	0.215
LE27	0.003 (1)	0.027 (10)	0.005 (2)	0.222 (86)	BDL	0.257
Mean_LE_	0.008 (2)	0.024 (6)	0.007 (2)	0.383 (90)	BDL	0.423
AA1	0.004 (5)	0.006 (9)	0.002 (3)	0.064 (84)	BDL	0.076
AA2	0.002 (3)	0.007 (10)	0.001 (2)	0.060 (85)	BDL	0.071
AA3	0.002 (5)	0.004 (10)	0.002 (5)	0.031 (80)	BDL	0.038
AA4	0.002 (7)	0.002 (5)	0.001 (5)	0.023 (82)	BDL	0.028
AA5	0.003 (5)	0.005 (8)	0.004 (6)	0.055 (82)	BDL	0.068
AA6	0.001 (3)	0.002 (3)	0.001 (3)	0.046 (91)	BDL	0.050
AA7	0.002 (7)	0.003 (8)	0.002 (5)	0.027 (81)	BDL	0.034
AA8	0.004 (8)	0.003 (7)	0.002 (3)	0.039 (82)	BDL	0.047
AA9	0.003 (5)	0.005 (9)	0.002 (3)	0.047 (83)	BDL	0.057
AA10	0.003 (7)	0.005 (11)	0.002 (5)	0.033 (78)	BDL	0.042
AA11	0.006 (12)	0.005 (10)	0.002 (4)	0.037 (74)	BDL	0.050
AA12	0.006 (18)	0.005 (14)	0.003 (8)	0.021 (60)	BDL	0.034
AA13	0.021 (24)	0.019 (22)	0.001 (1)	0.046 (53)	BDL	0.087
AA14	0.001 (4)	0.003 (11)	0.001 (2)	0.027 (83)	BDL	0.032
AA15	0.004 (13)	0.002(6)	0.001 (2)	0.023 (79)	BDL	0.029
Mean_AA_	0.004 (8)	0.005 (9)	0.002 (4)	0.039 (78)	BDL	0.050
CM1	0.006 (15)	BDL	BDL	0.034 (85)	BDL	0.040
CM2	0.005 (15)	BDL	BDL	0.030 (85)	BDL	0.036
CM3	0.009 (23)	BDL	BDL	0.032 (77)	BDL	0.041
CM4	0.009 (20)	BDL	BDL	0.037 (80)	BDL	0.047
Mean_CM_	0.007 (18)	BDL	BDL	0.033 (82)	BDL	0.041
AB1	1.316 (60)	0.052 (2)	0.238 (11)	0.585 (27)	BDL	2.192
AB2	1.305 (59)	0.032 (1)	0.126 (6)	0.750 (34)	BDL	2.212
AB3	2.140 (80)	0.046 (2)	0.132 (5)	0.360 (13)	BDL	2.677
AB4	1.144 (68)	0.050 (3)	0.187 (11)	0.299 (18)	BDL	1.680
Mean_AB_	1.476 (67)	0.045 (2)	0.171 (8)	0.499 (23)	BDL	2.190

Notes: (1) AsB, DMA^V^, MMA^V^, uAs, iAs, oAs, and total As are the abbreviations of arsenobetaine, dimethylarsenic acid, monomethylarsonic acid, unknown organic arsenic, inorganic arsenic (total), organic arsenic (total), and total arsenic, respectively; (2) Concentrations are presented as the means of thrice determinations with the relative standard deviation (RSD) of less than 7%; (3) BDL denotes the values below the detection limit.

**Table 2:** The ranges of sample values in the three-dimensional scatter plot (PC1 vs. PC2 vs. PC3).

Samples	PC1	PC2	PC3
WOS	1.40; 6.55	−0.60; 0.45	−0.10; 0.05
COS	−2.40; −2.30	0.45; 0.80	−0.10; 0.05
LE	−2.70; −1.50	−0.40; −0.05	−0.20; 0.00
AA	−2.80; −2.55	−0.15; 0.15	−0.15; 0.35
CM	−2.80; −2.60	−0.10; 0.00	0.00; 0.20
AB	−1.10; −0.05	−0.30; 0.00	0.55; 0.95

## Supplementary Materials

The following are available online. Figures S1–S5: Total ion currents of the standards adding tests (Figure S1: COS; Figure S2: CM; Figure S3: AB; Figure S4: LE; and Figure S5: AA), Table S1: Analytical performances for total As and As species analysis, Table S2: Precision of the methods, Table S3: Recovery of the methods, Table S4: Values of the National Standard Reference Materials (mg/kg, mean ± standard deviation) and determined values for total and inorganic As (*n* = 5), Table S5: Extraction efficiency of the method for As species analysis, Table S6: Concentrations of total As and percentages of As species in the samples of wild *O. sinensis*.

## Abbreviations

CFDA	China Food and Drug Administration
As	arsenic
iAs	inorganic arsenic
oAs	organic arsenic
As^III^	arsenite
As^V^	arsenate
uAs	unknown organic As
AsB	arsenobetaine
AsC	arsenocholine
MMA	monomethylarsonic acid
DMA	dimethylarsinic acid
ICP-MS	inductively coupled plasma mass spectroscopy
HPLC	high-performance liquid chromatography
WOS	wild *Ophiocordyceps sinensis*
COS	cultivated *Ophiocordyceps sinensis*
CM	*Cordyceps militaris*
AB	*Agaricus blazei*
LE	*Lentinus edodes*
AA	*Auricularia auricular*
PCA	principle component analysis
CRMs	certified reference materials
