# Supplementary material for: Novel Arsenic Markers for Discriminating Wild and Cultivated Cordyceps
Source: Molecules. 2018 Oct 29;23(11):2804. doi: 10.3390/molecules23112804 (PMC6278644; doi:10.3390/molecules23112804)
Supplement: Supplementary file 1 [file molecules-23-02804-s001.pdf]

Supplementary Tables and Figures

Novel arsenic markers for discriminating wild and cultivated *Cordyceps*

Lian-Xian Guo [1, †], Gui-Wei Zhang [2, †], Qing-Qing Li [1], Xiao-Ming Xu [3, *] and Jiang-Hai Wang [3, 4, *]

[1] Dongguan Key Laboratory of Environmental Medicine, School of Public Health, Guangdong Medical University, Dongguan, Guangdong 523808, China; glx525@gdmu.edu.cn

[2] Shenzhen Academy of Metrology and Quality Inspection, Shenzhen, Guangdong 518000, China; zhguiw98@163.com

[3] Guangdong Provincial Key Laboratory of Marine Resources and Coastal Engineering, School of Marine Sciences, Sun Yat-Sen University, Zhuhai 519082, China; wangjhai@mail.sysu.edu.cn; xuxiaom8@mail.sysu.edu.cn

[4] South China Sea Bioresource Exploitation and Utilization Collaborative Innovation Center, School of Marine Sciences, Sun Yat-Sen University, Guangzhou 510006, China; wangjhai@mail.sysu.edu.cn

* Authors to whom correspondence should be addressed. E-mails: wangjhai@mail.sysu.edu.cn (J.-H. Wang); xuxiaom8@mail.sysu.edu.cn (X.-M. Xu). Tel.: +86 20 39332212; Fax: +86 20 85261499.

[†] These authors contributed equally to this work.

List of Supplementary Tables and Figures

Table S1. Analytical performances for total arsenic and arsenic species analysis

Table 2S. Precision of the methods

Table 3S. Recovery of the methods

Table 4S. Values of the National Standard Reference Materials (mg/kg, mean standard deviation) and determined values for total and inorganic arsenic (n = 5)

Table 5S. Extraction efficiency of the method for arsenic species analysis

Table 6S. Concentrations of total As and percentages of arsenic species in the samples of wild *O. sinensis*

Figure 1S. Total ion currents of the standards adding tests for COS

Figure 2S. Total ion currents of the standards adding tests for CM

Figure 3S. Total ion currents of the standards adding tests for AB

Figure 4S. Total ion currents of the standards adding tests for LE

Figure 5S. Total ion currents of the standards adding tests for AA

Table 1S. Analytical performances for total arsenic and arsenic species analysis

**Analytes**	**Linear range (μg/L)**	**Linear equation**	***R***	**LOD (μg/kg)**	**LOQ (μg/kg)**
Total As	0.5 500	*y* = 2759.45 *x* + 6.55	0.9999	2.0	6.0
AsB	0.2 300	*y* = 17526.0 *x* + 491.6	1.0000	1.2	3.6
DMA^{V}	0.2 300	*y* = 19528.7 *x* + 391.9	0.9999	1.3	4.0
As^{III}	0.2 300	*y* = 17673.6 *x* + 5393.4	1.0000	1.0	3.0
MMA^{V}	0.5 300	*y* = 16420.9 *x*	0.9997	2.1	6.3
As^{V}	0.2 300	y = 14292.3 *x* + 10090.0	0.9997	1.3	4.0

Table 2S. Precision of the methods

Sample	As species	1	2	3	4	5	6	Average	RSD (%)
1	tAs	0.35	0.36	0.36	0.37	0.35	0.38	0.36	3.25
	AsB	0.0061	0.0063	0.0057	0.0064	0.0066	0.0067	0.0063	5.77
	DMA	0.015	0.018	0.017	0.017	0.018	0.018	0.017	6.81
	As(III)	0.18	0.20	0.19	0.18	0.19	0.20	0.19	4.71
	MMA	0.0085	0.0088	0.0078	0.0079	0.0083	0.0084	0.0083	4.54
	As(V)	0.16	0.15	0.15	0.15	0.15	0.16	0.15	3.37
2	tAs	1.23	1.25	1.16	1.18	1.26	1.17	1.21	3.60
	AsB	0.017	0.018	0.020	0.018	0.017	0.018	0.018	6.09
	DMA	0.064	0.062	0.058	0.061	0.064	0.066	0.063	4.50
	As(III)	0.72	0.70	0.66	0.69	0.68	0.68	0.69	2.97
	MMA	0.014	0.014	0.016	0.015	0.015	0.015	0.015	5.07
	As(V)	0.41	0.40	0.44	0.41	0.40	0.43	0.42	3.96

Table 3S. Recovery of the methods

Analytes	Back ground value (μg/L)	Added value (μg/L)	Measured value (μg/L)	Recovery (%)
tAs	21.30	5.00	26.13	96.60
		20.00	42.22	105.00
		50.00	70.51	98.40
AsB	0.329	5.00	5.46	103.00
		10.00	9.98	96.60
		20.00	19.45	95.70
DMA^{V}	0.88	5.00	6.11	105.00
		10.00	10.20	93.80
		20.00	21.51	103.00
As^{III}	10.00	5.00	14.60	92.00
		10.00	19.70	98.50
		20.00	28.44	94.80
MMA^{V}	0.434	5.00	5.25	96.30
		10.00	10.80	103.50
		20.00	19.88	97.30
As^{V}	7.88	5.00	13.10	104.00
		10.00	18.30	102.30
		20.00	29.55	106.00

Table 4S. Values of the National Standard Reference Materials (mg/kg, mean   standard deviation) and determined values for total and inorganic arsenic (*n* = 5)

Sample type	Reference materials	Certified value (mg/kg)	Determined value (mg/kg)	Recovery (%)
Green Chinese onion	GBW10049	0.52 0.11	0.511 0.06	98.3
Pork liver	GBW10051	1.40 0.30	1.38 0.17	98.6
Yellow- fin tuna	GBW08573	5.08 0.39	5.13 0.15	101.0
Rice	GBW100358	0.16 0.02 (total As)	0.163 0.014	102.0
		0.13 0.02 (iAs)	0.140 0.007	108.0

Table 5S. Extraction efficiency of the method for arsenic species analysis

Sample Nos.	tAs[1] (D HNO_3)	tAs[2] (C HNO_3)	Extraction efficiency[3] (%)	Sample Nos.	tAs (D HNO_3)	tAs (C HNO_3)	Extraction efficiency (%)
1	0.37	0.36	104.2	11	0.62	0.64	96.5
2	0.28	0.31	91.5	12	0.44	0.50	88.0
3	0.29	0.31	94.3	13	0.60	0.69	87.0
4	0.39	0.4	96.9	14	0.30	0.31	97.0
5	0.42	0.44	95.2	15	0.45	0.47	95.1
6	0.34	0.35	98.4	16	1.03	1.05	98.0
7	1.20	1.16	103.7	17	0.31	0.34	92.6
8	0.30	0.33	89.8	18	0.43	0.47	91.6
9	0.28	0.32	87.5	19	0.43	0.47	91.9
10	0.35	0.36	98.5	20	0.51	0.54	94.5

[1] The concentrations of total As determined by ICP-MS in the extraction which digested by dilute HNO_3 (0.15 mol/L).

[2] The concentrations of total As determined by ICP-MS in the extraction which digested by concentrated HNO_3.

[3] The percentages of tAs[1] compared to tAs[2], showing the extraction effects for arsenic species analysis.

Table 6S. Concentrations of total As and percentages of arsenic species in the samples of wild *O. sinensis*

Samples*	AsB (%)	DMA (%)	MMA (%)	iAs (%)	uAs (%)	tAs (mg/kg)
WOS1	2	0	0	7	91.2	4.76
WOS2	2	0	0	6	91.8	5.00
WOS3	2	0	0	8	90.7	4.09
WOS4	2	0	0	8	90.4	4.15
WOS5	2	0	0	8	89.6	4.69
WOS6	2	0	0	7	90.6	5.08
WOS7	0	0	0	1	99.0	5.32
WOS8	0	0	0	0	100.0	9.18
WOS9	0	0	0	0	100.0	6.61
WOS10	0	0	0	0	100.0	7.19
WOS11	0	0	0	0	100.0	8.91
WOS12	0	0	0	1	99.0	4.85
WOS13	0	0	0	0	100.0	5.86
WOS14	0	0	0	1	99.0	5.74
WOS15	0	0	0	0	100	7.84
WOS16	0	0	0	0	100	5.44
WOS17	0	0	0	1	99.0.0	7.34
WOS18	0	0	0	0	100.0	6.06
WOS19	0	0	0	1	99.0	6.32
WOS20	0	0	0	0	100.0	6.78
WOS21	0	0	0	0	100.0	6.33
WOS22	0	0	0	1	99.0	5.63
WOS23	0	0	0	0	100.0	6.57
WOS24	0	0	0	0	100.0	6.05
WOS25	0	0	0	0	100.0	5.77
WOS26	0	0	0	0	100.0	4.71
WOS27	0	0	0	1	99.0	4.76
WOS28	0	0	0	0	100.0	5.92
WOS29	0	0	0	0	100.0	5.19
WOS30	0	0	0	1	99.0	5.46
WOS31	0	0	0	1	99.0	5.72
WOS32	0	0	0	0	100.0	5.78
WOS33	0	0	0	1	99.0	7.56
WOS34	0	0	0	1	99.0	8.02
WOS35	0	0	0	0	100.0	7.46

WOS36	0	0	0	1	99.0	5.32
WOS37	0	0	0	1	99.0	6.12
WOS38	0	0	0	1	99.0	4.36

*The data of WOS 1 5 and WOS 6 38 were cited from Guo et al. (2018) and Cao et al. (2015), respectively.

References

1. Guo, L.X.; Zhang, G.W.; Wang, J.T.; Zhong, Y.P.; Huang, Z.G. Determination of As species in *Ophiocordyceps sinensis* from major habitats in China by HPLC-ICP-MS and the edible hazard assessment. *Molecules.* **2018**, *23*, 1012.
2. Cao, X.; Wang, J.; Li, J.M.; Wang, S.Z. Analysis of arsenic speciation in *Cordyceps sinensis* in Tibet by HPLC-HG-AFS. *Chinese Tradit. Patent Med.* **2015**, *37*, 1985 1989.

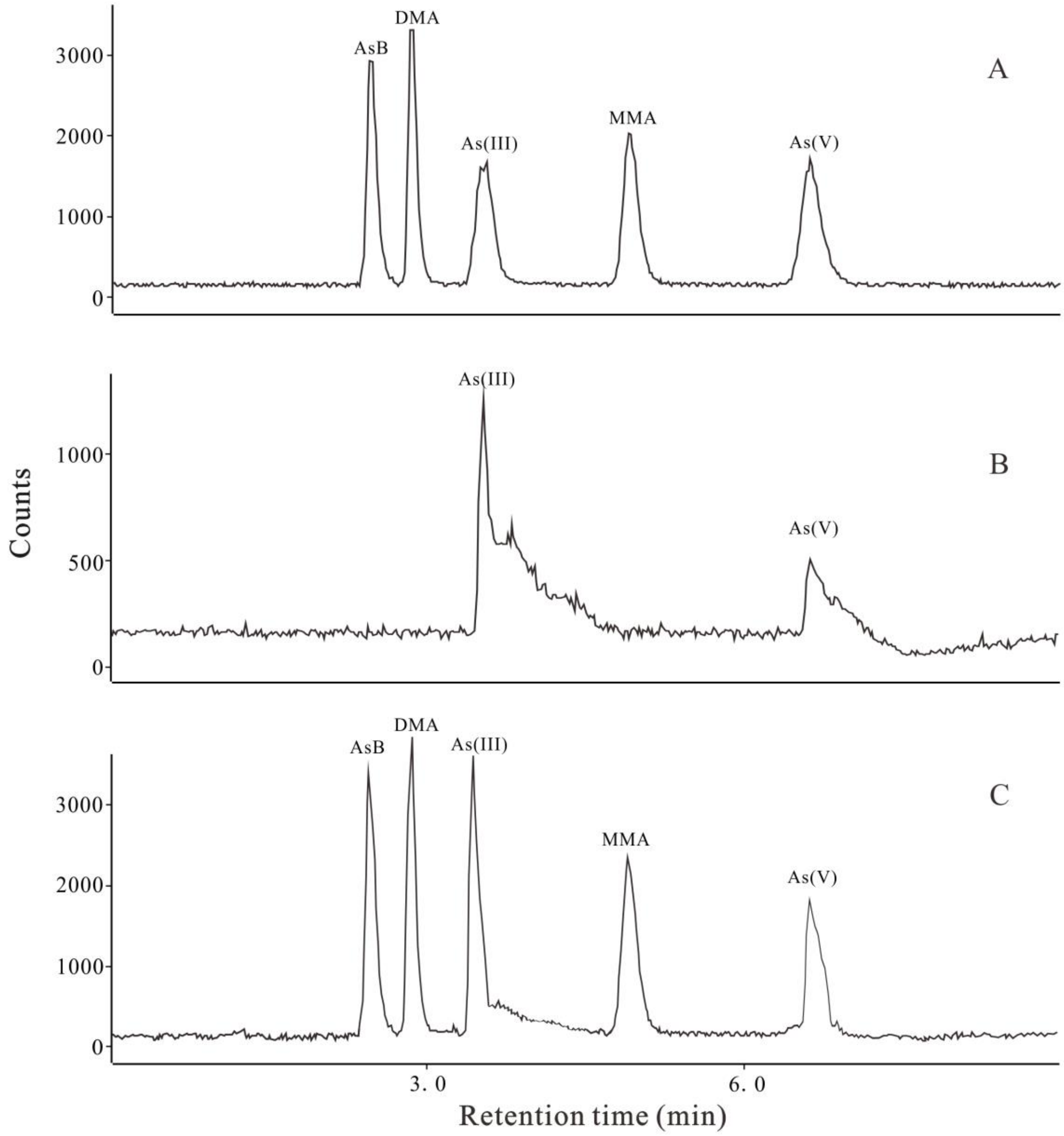

Figure 1S. Total ion currents of the standards adding tests for COS

A: 2.5 ppb standards; B: COS; and C: 2.5 ppb standards added in COS.

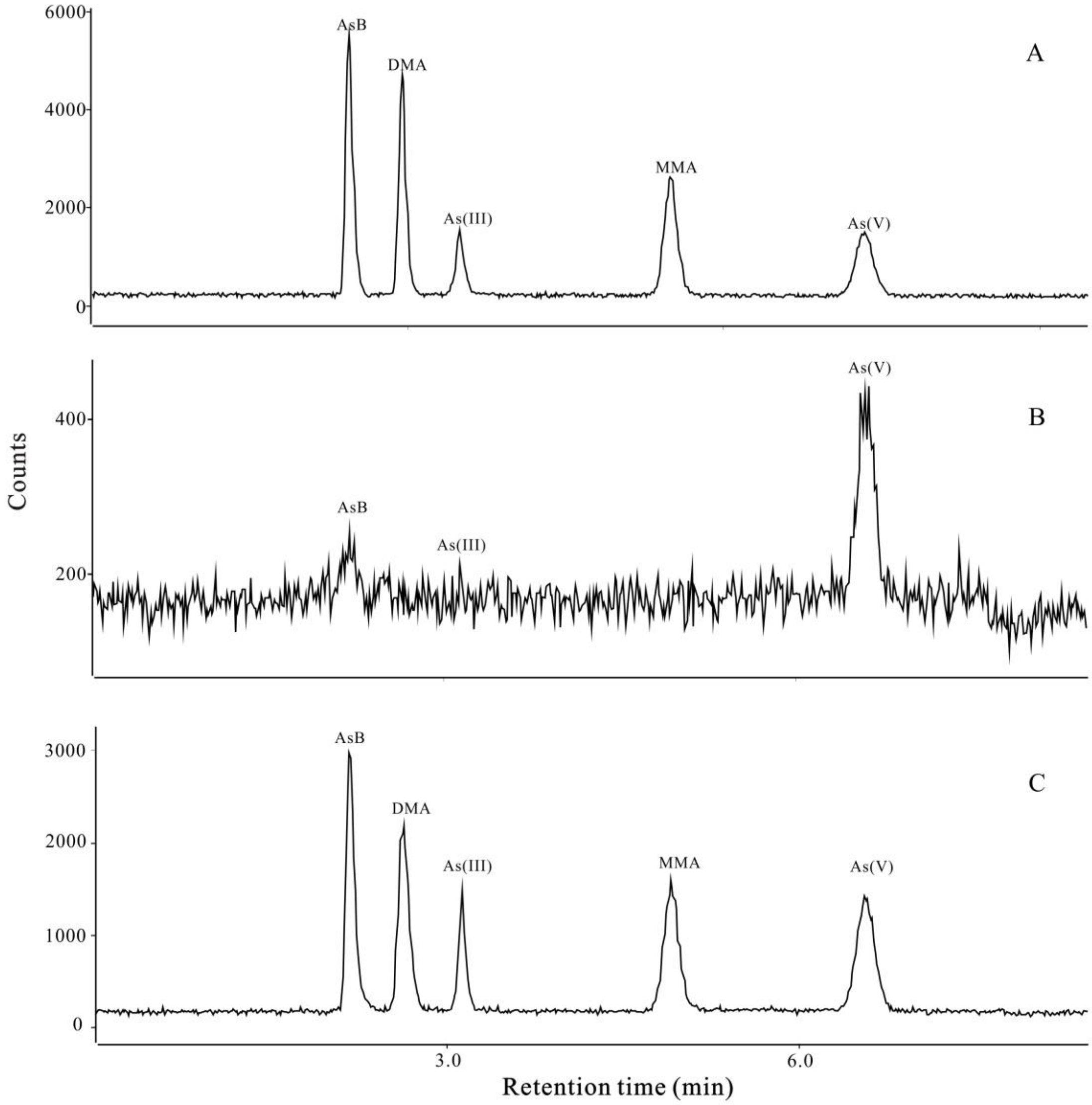

Figure 2S. Total ion currents of the standards adding tests for CM

A: 5 ppb standards; B: CM; and C: 5 ppb standards added in CM.

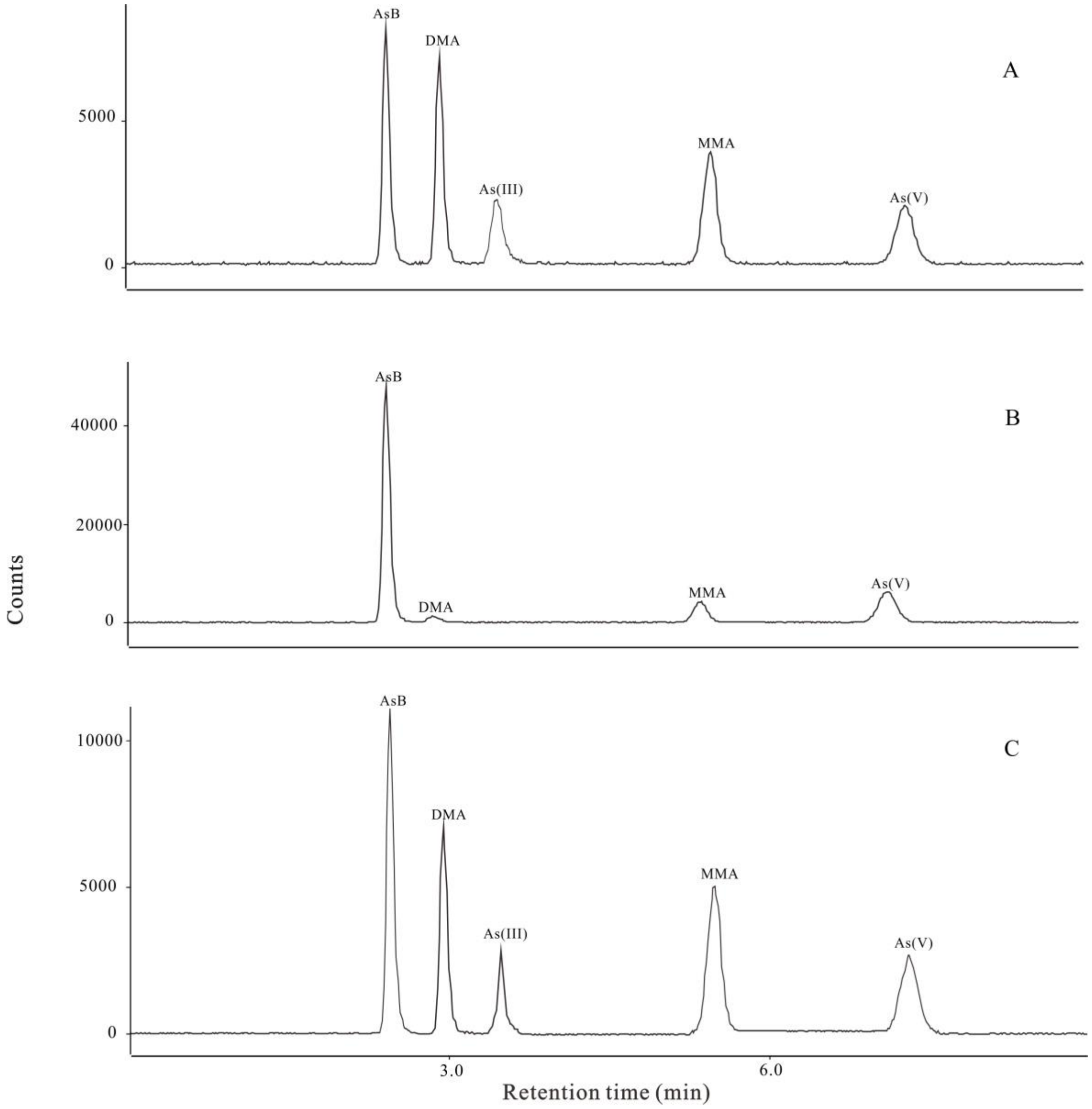

Figure 3S. Total ion currents of the standards adding tests for AB

A: 10 ppb standards; B: AB; and C: 100 ppb standards added in AB and then diluted to 1:10.

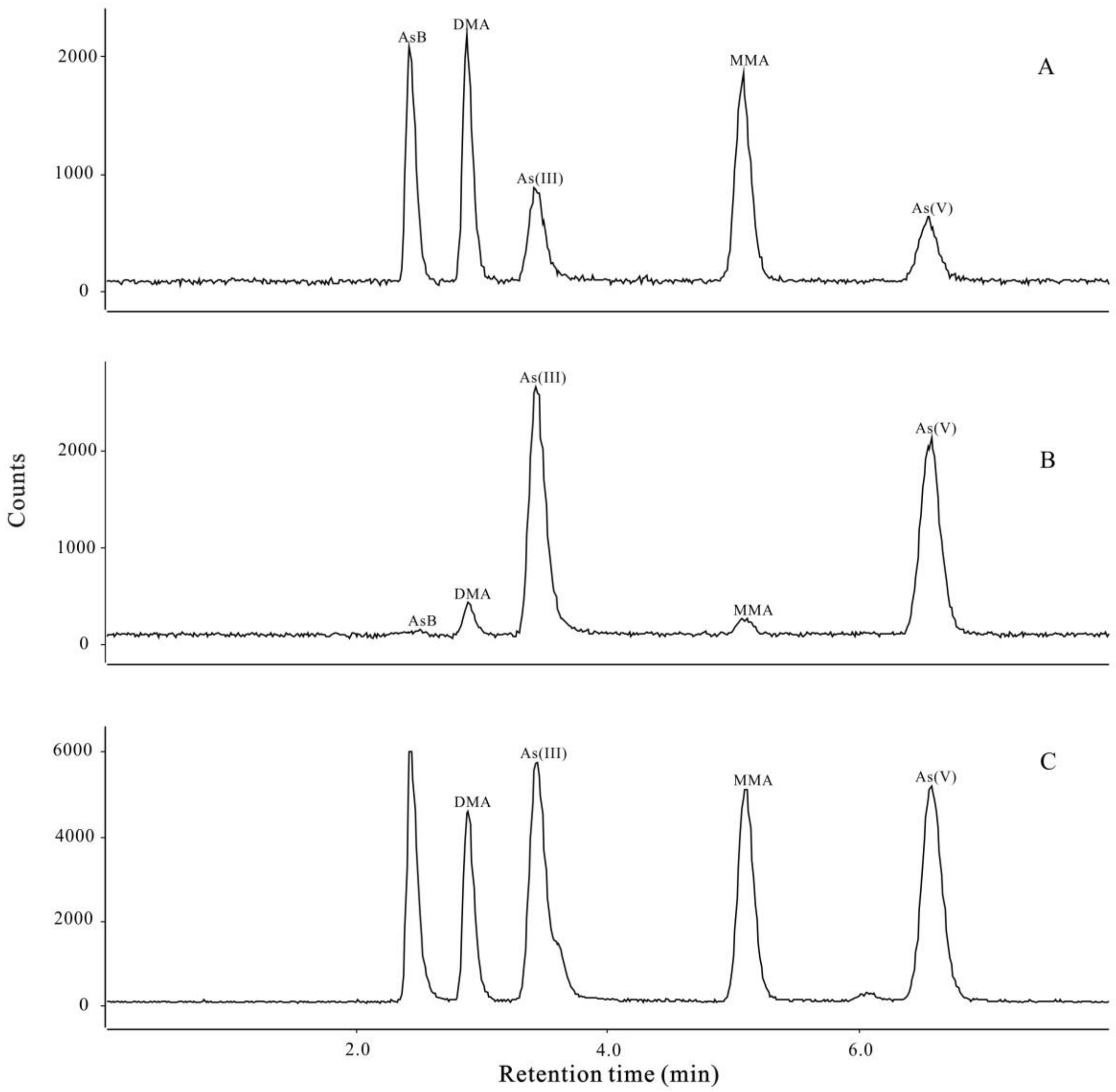

Figure 4S. Total ion currents of the standards adding tests for LE

A: 5 ppb standards; B: LE; and C: 15 ppb standards added in LE.

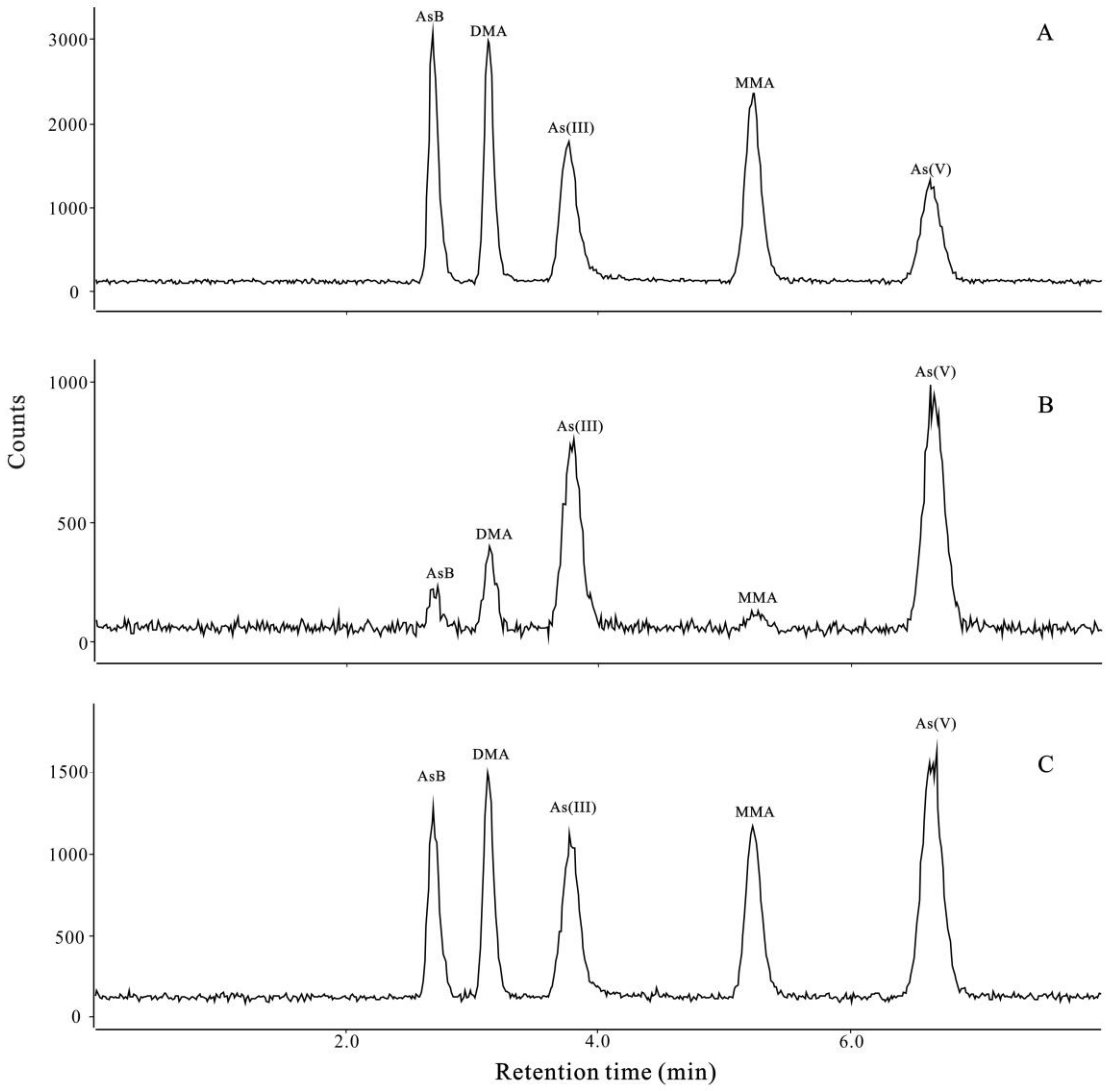

Figure 5S. Total ion currents of the standards adding tests for AA

A: 5 ppb standards; B: AA; and C: 2 ppb standards added in AA.
